# Ionic Liquid-Based Electrolyte Membranes for Medium-High Temperature Lithium Polymer Batteries

**DOI:** 10.3390/membranes8030041

**Published:** 2018-07-10

**Authors:** Guk-Tae Kim, Stefano Passerini, Maria Carewska, Giovanni Battista Appetecchi

**Affiliations:** 1 Helmholtz Institute Ulm-Karlsruhe Institute of Technology, Helmholtzstrasse 11, 89081 Ulm, Germany; guk-tae.kim@kit.edu (G.-T.K.); stefano.passerini@kit.edu (S.P.); 2 Karlsruher Institute of Technology (KIT), P.O. Box 3640, 76021 Eggenstein-Leopoldshafen, Germany; 3 ENEA, Agency for New Technologies, Energy and Sustainable Economic Development, DTE-PCU-SPCT, Via Anguillarese 301, 00123 Rome, Italy; maria.carewska@enea.it; 4 ENEA, Agency for New Technologies, Energy and Sustainable Economic Development, SSPT-PROMAS-MATPRO, Via Anguillarese 301, 00123 Rome, Italy

**Keywords:** ionic liquids, *N*-butyl-*N*-methylpyrrolidinium bis(trifluoromethanesulfonyl)imide, poly(ethyleneoxide), polymer electrolytes, lithium polymer batteries

## Abstract

Li^+^-conducting polyethylene oxide-based membranes incorporating *N*-butyl-*N*-methylpyrrolidinium bis(trifluoromethanesulfonyl)imide are used as electrolyte separators for all-solid-state lithium polymer batteries operating at medium-high temperatures. The incorporation of the ionic liquid remarkably improves the thermal, ion-transport and interfacial properties of the polymer electrolyte, which, in combination with the wide electrochemical stability even at medium-high temperatures, allows high current rates without any appreciable lithium anode degradation. Battery tests carried out at 80 °C have shown excellent cycling performance and capacity retention, even at high rates, which are never tackled by ionic liquid-free polymer electrolytes. No dendrite growth onto the lithium metal anode was observed.

## 1. Introduction

Large-scale applications such as automotive, stationary, deep-sea drilling devices need batteries to be capable of operating safely at medium-high temperatures with very good performance and cycle life; i.e., without appreciable degradation phenomena. In addition, even devices generally operating around room temperature could be accidentally subjected to prolonged overheating, thus requiring high thermal stability. In this scenario, electrolytes play a key role.

Rechargeable lithium batteries are an excellent choice as advanced electrochemical energy storage systems due to their high energy density and cycle life [1,2]. Recently published manuscripts report that ion conducting polymer membranes, realized through common materials and up-scalable processes, can act as electrolyte separators for rechargeable lithium battery systems. J.R. Nair et al. [3] have prepared methacrylic-based PEs, reinforced with both cellulose hand-sheets and nanosize cellulose fibers, by UV-induced free radical photo-polymerization. Similarly, rigid–flexible composite electrolyte membranes, based on poly(ethyl *α*-cyanoacrylate) and cellulose backbone, have been prepared through an in-situ polymerization process by P. Hu et al. [4]. These cross-linking techniques, also successfully proposed for Na^+^ conducting PEs [5], have shown short processing times, easy up-scalability and eco-compatibility, and have enabled gel polymer electrolytes (GPEs) with wide electrochemical stability windows and high room temperature ionic conductivity in combination with good mechanical properties to be obtained. Poly(vinylidene difluoride)-based GPEs were obtained via the phase-inversion method [6]. The use of nano-clay filler and pore-forming agent, i.e., poly(vinylpyrrolidone), was seen to significantly improve the electrolyte uptake and the ion transport properties. H. Li et al. [7] have combined the advantages of GPEs with those of ceramic conductors to prepare sandwiched structure composite electrolytes with enhanced electrochemical performance. Reviews of GPE systems, addressed to Li/S [8] and Li-ion [9] battery systems, were recently published.

However, commercial lithium-ion batteries, even employing GPEs, do not behave well at medium-high temperatures as the organic electrolyte quickly degrades above 50 °C, thus irreversibly ageing the electrochemical device [10,11,12]. In this scenario, the development of solvent-free polymer electrolytes is undoubtedly appealing from safety and engineering points of view and opens new perspectives to applications in electrochemical devices [8,9,13,14,15,16,17]. In addition, polymer electrolytes (PEs) can be easily and cheaply manufactured into low thicknesses (<100 μm) and shapes not allowed for supported liquid electrolytes, offering a new concept of solvent-free, all-solid-state, thin-layer, flexible (both mechanically and in design), robust, lithium polymer batteries (LPBs). Finally, PEs play a second role in composite electrodes as binders and ionic conductors [18].

Nevertheless, the realization of all-solid-state lithium battery systems has been prevented so far by the low ionic conductivity of PEs, especially at ambient temperature. For instance, poly(ethyleneoxide)-lithium salt (PEO-LiX) complexes, considered to be very good candidates as electrolyte separators for LPBs [13,14,15,16,17,18,19,20,21,22,23], approach conduction values of interest for practical applications (>10^−4^ S·cm^−1^) only above 70 °C, i.e., when the polymer is in the amorphous state [13,14,17,20,21,22]. However, even at medium-high temperatures (≥90 °C) LPBs exhibit high performance only at low current rates (≤0.1C) [18,22,23], thus preventing applications requiring high power density.

An appealing way to overcome the conductivity drawback is represented by the incorporation of ionic liquids (ILs) into the polymer electrolytes [24]. ILs, i.e., salts which are molten at room temperature consisting of organic cations and inorganic/organic anions [25,26,27], display several peculiarities such as their extremely low flammability, negligible vapor pressure, high chemical–electrochemical–thermal stability, fast ion transport properties, good power solvency and high specific heat. In the last years, it was successfully demonstrated [24,28,29,30,31,32,33,34] how the addition of ILs to PEO-based electrolytes enhances the ionic conductivity above 10^−4^ S·cm^−1^ at 20 °C—i.e., more than two orders of magnitude higher than that of ionic liquid-free PEs—allowing LPBs to obtain a significant cycling performance at near room temperature (30–40 °C) [24,29,30,31,32,33,34].

In the present work, we show how the incorporation of ionic liquids improves the performance of PEO-based electrolytes even at medium-high temperatures, especially at high current rates, without any evident material degradation and battery cycle life depletion, making the IL-containing PEO membrane an appealing electrolyte separator for LIBs operating at medium-high temperatures. *N*-butyl-*N*-methylpyrrolidinium bis(trifluoromethanesulfonyl)imide (PYR_14_TFSI) was selected as the ionic liquid [24].

## 2. Materials and Methods

### 2.1. Synthesis of the Ionic Liquid

The PYR_14_TFSI ionic liquid was synthesized through an eco-friend route, reported in detail elsewhere [35,36].

### 2.2. Preparation of the Polymer Electrolyte and the Composite Cathode

The ionic liquid-based polymer electrolyte and composite cathode were prepared through a solvent-free process [33] carried out in a very low relative humidity dry-room (R.H. < 0.1% at 20 °C). The material components, i.e., PEO (Dow Chemical, Midland, MI, USA, WSR 301, M_W_ = 4,000,000 a.u.), lithium bis(trifluoromethanesulfonyl)imide (LiTFSI, 3M, battery grade) and PYR_14_TFSI, were vacuum dried at 50 °C for 48 h (PEO) and at 120 °C for 24 h (lithium salt and ionic liquid). PEO and LiTFSI (EO:Li mole ratio = 1:0.1) were intimately mixed in a mortar, and then PYR_14_TFSI was added to achieve a (PYR_14_)^+^/Li^+^ mole ratio equal to 1:1. In previous papers [24,33], we have shown that this ratio represents a good compromise between ion transport properties and interfacial stability. The P(EO)_1_(LiTFSI)_0.1_(PYR_14_TFSI)_0.1_ past-like electrolyte blend was annealed under vacuum at 100 °C overnight in order to allow the full diffusion of the lithium salt and ionic liquid through the PEO host, therefore obtaining a homogeneous mixture. Finally, the so-obtained rubber-like material was hot-pressed at 100 °C for 2 min to form 70–80 μm thick films. Ionic liquid-free, P(EO)_1_(LiTFSI)_0.1_ binary polymer electrolytes were prepared for comparison purposes.

The cathode tape was prepared by intimately blending LiFePO_4_ active material (Sud Chemie, Munich, Germany) and KJB carbon (electronic conductor, Akzo Nobel, Amsterdam, The Netherlands). LiFePO_4_ and KJB were previously vacuum dried at 120 °C for at least 24 h. Separately, PEO, LiTFSI and PYR_14_TFSI were roughly mixed (to obtain a paste-like mixture) and then added to the LiFePO_4_-KJB blend. The resulting cathodic mixture was firstly annealed at 100 °C overnight and then hot-pressed to form preliminary films (200–300 μm thick) which were cold-rolled to obtain the final cathode tape (<50 μm) and to remove any porosity within the composite cathode [37]. Finally, 12 mm diameter cathode discs (active area equal to 1.13 cm^2^) were punched for the battery tests. The active material mass loading ranged from 4 to 5 mg·cm^−2^, corresponding (accounting for a theoretical capacity of LiFePO_4_ equal to 170 mA·h·g^−1^) to a capacity from 0.7 to 0.8 mA·h·cm^−2^.

### 2.3. Thermal Analysis

DSC measurements were run using a differential scanning calorimeter (TA Instruments, model Q100, New Castle, DE, USA). The samples, upon housing (within the dry room) in sealed Al pans, were cooled (10 °C·min^−1^) from room temperature down to −140 °C and then heated (10 °C·min^−1^) up to 150 °C.

The thermal stability was verified in a nitrogen atmosphere through TG analysis carried out by a SDT 2960 equipment, simultaneous TG-DTA (TA Instruments, New Castle, DE, USA) with Thermal Solution Software (version 1.4, Thermal Solutions Inc, Ann Arbor, MI, USA). During the experiments, the atmosphere above the samples was fixed by flowing high purity nitrogen atmosphere at a flow rate of 100 mL·min^−1^. The experiments were performed on 5–10 mg samples (handled in the dry room), which were housed in platinum crucibles. The thermal stability was initially investigated by running a heating scan from room temperature up to 500 °C at a scan rate of 10 °C·min^−1^.

### 2.4. Cell Assembly

The electrochemical measurements on the polymer electrolyte samples were carried out on two-electrode cells fabricated in the dry room. Two different cell types (active area equal from 2 to 3 cm^2^) were assembled by sandwiching a polymer electrolyte separator between (i) two Li foil electrodes (50 μm thick, supported onto Cu grids as the current collectors) for determining, respectively, the resistance at the interface with the lithium anode and the limiting diffusion current density; (ii) a nickel foil (working electrode, 100 μm thick, used also as the current collector) and a lithium foil (counter electrode, 50 μm thick, supported onto a Cu grid as the current collector) for the linear sweep voltammetry tests. In the latter kind of cell, a tiny lithium strip (50 μm thick, supported onto a Ni grid as the current collector) was used as the reference electrode.

The electronic conductivity of the ionic liquid-containing LiFePO_4_ composite cathode was investigated as a function of the carbon content by carrying out impedance measurements on symmetrical Al/cathode/Al cells. The composite cathode tape was interlayered between two Al foils (20 μm thick), which were also used as the current collectors.

The solid-state Li/LiFePO_4_ batteries (cathode limited) were fabricated (inside the dry room) by laminating a lithium foil (50 μm thick), a P(EO)_10_(LiTFSI)_0.1_(PYR_14_TFSI)_0.1_ polymer electrolyte separator and a LiFePO_4_-based composite cathode tape (plated onto a 20 μm thick Al foil). Aluminum and copper grids were used as the cathodic and anodic current collector, respectively. The electrochemically active area of the Li/LiFePO_4_ cells was 1.13 cm^2^.

All assembled cells were housed in soft envelopes, evacuated for at least 1 h (10^−2^ mbar) and then vacuum-sealed. Finally, the cells were laminated twice by hot-rolling at 100 °C to improve the electrolyte/electrode interfacial contact.

### 2.5. Electrochemical Tests

Impedance measurements were performed on symmetrical Li/polymer electrolyte/Li (frequency range: 65 kHz–10 mHz; temperature range: 20–80 °C) and Al/composite cathode/Al (10 kHz–1 Hz, 20 °C) cells by a Frequency Response Analyzer, F.R.A. (Schlumberger Solartron, mod. 1260, Leicester, UK). The analysis of the AC responses was carried out by an equivalent circuit model taking into account all possible contributes to the impedance of the cell under test [38]. The validity of the selected circuit was confirmed by fitting the AC responses using a non-linear least-square (NLLSQ) software developed by Boukamp [39,40] (only fits characterized by a χ^2^ factor lower than 10^−4^ were considerable acceptable [39,40]).

The electrochemical stability window (ESW) of the P(EO)_1_(LiTFSI)_0.1_(PYR_14_TFSI)_0.1_ polymer electrolyte was evaluated by linear sweep voltammetries (LSVs) run at 0.5 mV·s^−1^ in the 20–80 °C temperature range. The measurements were performed by scanning the cell potential from the open circuit value (OCV) towards more negative or positive potentials to determine the cathodic and anodic electrochemical stability limits, respectively. The LSVs were performed at least twice on each electrolyte to confirm the results obtained, using fresh samples and clean electrodes for each test. The measurements were performed at 20 °C using an Electrochemical Interface (Schlumberger Solartron, mod. 1287, Leicester, UK).

The limiting diffusion current density of the P(EO)_1_(LiTFSI)_0.1_ and P(EO)_1_(LiTFSI)_0.1_(PYR_14_TFSI)_0.1_ polymer electrolytes was determined by potentiodynamic measurements on symmetrical Li/electrolyte/Li cells, i.e., the cell voltage was linearly increased from the OCV value (a few mV) at a scan rate of 0.01 mV·s^−1^ until the current response achieves a steady state. The measurements were performed at temperatures ranging from 40 to 80 °C by a potentiostat/galvanostat (MACCOR, mod. 4000, Tulsa, OK, USA).

The cycling performance of the Li/LiFePO_4_ polymer cells was evaluated under charge/discharge rates ranging from 0.1C (*j* = 0.07–0.08 mA·cm^−2^) to 1C (*j* = 0.7–0.8 mA·cm^−2^) at 80 °C. The battery tests were performed using a multiple battery tester (MACCOR, mod. S4000, Tulsa, OK, USA). The voltage cut-offs were fixed at 4.0 V (charge step) and 2.0 V (discharge step), respectively. During the experiments, the cells were held in a climatic chamber (Binder GmbH, mod. MK53, Tuttlingen, Germany) with a temperature control of ±0.1 °C.

## 3. Results and Discussion

### 3.1. Ionic Liquid-Based Polymer Electrolytes

The solvent-free procedure allowed homogeneous, freestanding, polymer electrolyte membranes with good mechanical properties to be obtained. In addition, the ionic liquid-containing P(EO)_1_(LiTFSI)_0.1_(PYR_14_TFSI)_0.1_ sample looks rather sticky, thus resulting (even if not easily handled) in improved contact at the interface with electrodes.

**Figure 1:**
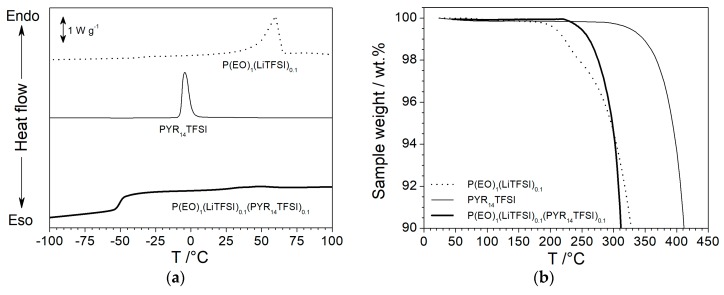
DSC (panel **a**) and TGA (panel **b**) traces of P(EO)_10_(LiTFSI)_1_ and P(EO)_10_(LiTFSI)_1_(PYR_14_TFSI)_1_ polymer electrolyte samples. Scan rate: 10 °C·min^−1^. The PYR_14_TFSI ionic liquid is reported for comparison purposes.

The results of the DSC investigation are illustrated in Figure 1a. The P(EO)_1_(LiTFSI)_0.1_ electrolyte shows a broad endothermic melting peak centered around 60 °C [21,41] and a weak glass transition (T_g_) feature located at −39 °C. The pure PYR_14_TFSI ionic liquid, reported for comparison purposes, exhibits only a melting peak around −7 °C [42]; i.e., the absence of glass transition and exothermal “cold crystallization” features suggest that the IL sample was fully crystallized prior to running the DSC measurements [43]. The incorporation of PYR_14_TFSI into the P(EO)_1_(LiTFSI)_0.1_ electrolyte results in almost complete disappearance of the melting peak in the DSC trace, which displays only the T_g_ feature around −55 °C, clearly indicating that the P(EO)_1_(LiTFSI)_0.1_(PYR_14_TFSI)_0.1_ electrolyte is amorphous even at room temperature.

The thermal stability is a mandatory requirement for electrolytes to be addressed to battery systems for medium-high temperature applications. Figure 1b compares the TGA trace (in nitrogen atmosphere) of the P(EO)_1_(LiTFSI)_0.1_ and P(EO)_1_(LiTFSI)_0.1_(PYR_14_TFSI)_0.1_ electrolyte membranes. The IL-free sample exhibits a weight loss above 180 °C, whereas the addition of the ionic liquid component results in thermal stability increase up to 220 °C. It should be noted that PYR_14_TFSI is seen to be thermally stable up 290 °C. Therefore, we can reasonably hypothesize that the ionic liquid, properly incorporated within the polymer host, is able to protect the PEO chains by thermal degradation. Something similar was previously observed in other PEO electrolytes [41], in which the IL agent, suitably dispersed through the polymeric matrix, was seen to prevent the oxidation of the polymer host above 4 V (vs. Li^+^/Li°).

The effect of the incorporation of the PYR_14_TFSI ionic liquid on the ion transport properties of the polymer electrolyte is summarized in Table 1. A remarkable conductivity increase is observed, especially at ambient temperature and below. For instance, the P(EO)_1_(LiTFSI)_0.1_(PYR_14_TFSI)_0.1_ sample shows ion conduction values three and two orders of magnitude higher than that of the IL-free sample at −20 °C and 20 °C [31,33], respectively. More than 10^−4^ S·cm^−1^ are exhibited at 20 °C, this is of interest for applications in practical devices and commonly not approached in polymer electrolyte membranes. These results support faster ion conduction through the PEO electrolyte due both to a much larger content of the amorphous phase, in agreement with the DSC data of Figure 1a, and to the enhanced mobility of the Li^+^ cations resulting from the presence of PYR_14_TFSI; i.e., the addition of ionic liquid results in large anion excess with respect to the lithium cations. Therefore, the strength of the Li^+^⋯Anion^−^ interaction reduces the role of the PEO chains in the coordination of the lithium cations, e.g., as a result from the competition with the PEO⋯Li^+^ interactions [24]. At medium-high temperatures, the conductivity of the P(EO)_1_(LiTFSI)_0.1_(PYR_14_TFSI)_0.1_ electrolyte is seen to approach or exceed 10^−3^ S·cm^−1^, still displaying a substantial rise with respect to that of the binary IL-free P(EO)_1_(LiTFSI)_0.1_ [31,33].

**Figure 2:**
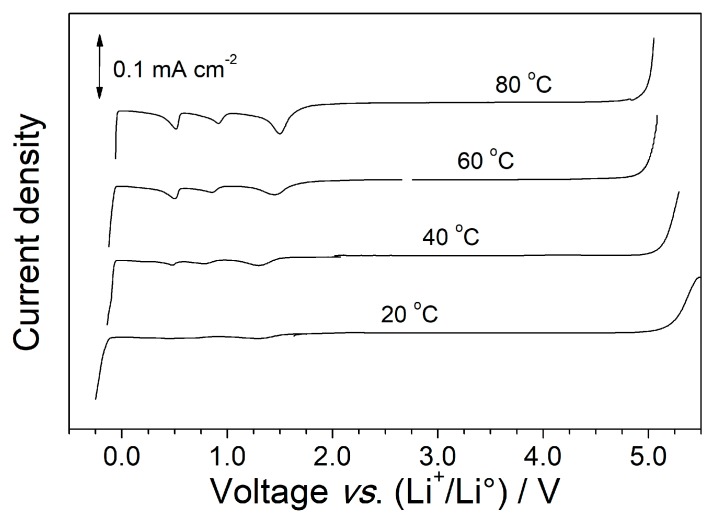
Electrochemical stability window of the P(EO)_1_(LiTFSI)_0.1_(PYR_14_TFSI)_0.1_ polymer electrolyte sample at different operating temperatures. Nickel is used as the working electrode, lithium as counter and reference electrodes. Scan rate: 0.5 mV·s^−1^.

An important requirement for any electrolyte is its capacity to successfully and efficiently allow electrode reactions, at the operating temperature of the device, without appreciable electrochemical degradation (oxidation/reduction) phenomena. Therefore, the electrochemical stability window (ESW) of the P(EO)_1_(LiTFSI)_0.1_(PYR_14_TFSI)_0.1_ electrolyte system was investigated as a function of the temperature. The results, reported in Figure 2 as linear sweep voltammetry curves, evince only a moderate, even if progressive, reduction of the ESW on passing from 20 to 80 °C. In particular, the anodic stability (related to oxidation processes of the electrolyte) detected at 80 °C differs by just 200 mV with respect to that recorded at 20 °C. Conversely, no practical variation is observed on the cathodic side with the temperature increase, displaying massive electrolyte reduction well below 0 V vs. Li^+^/Li°, which allows lithium plating also at 80 °C. A very low current flow (<25 μA·cm^−2^) is observed up to the anodic breakdown voltage, thus supporting the high purity of the P(EO)_1_(LiTFSI)_0.1_(PYR_14_TFSI)_0.1_ sample. On the cathodic verse, three weak (≤20 µA·cm^−2^) features, progressively evinced with the temperature increase, are observed around 1.5 V, 0.9 V and 0.5 V vs. Li^+^/Li°, respectively. Results previously reported in the literature [44] suggest that the peaks located at 1.5 V and 0.5 V vs. Li^+^/Li° are ascribable to the Li^+^ cation intercalation process into the native Ni_x_O film onto the nickel working electrode surface, whereas the feature at 0.9 V is likely due to impurities, i.e., probably water [45]. To summarize, the P(EO)_1_(LiTFSI)_0.1_(PYR_14_TFSI)_0.1_ electrolyte is allowed to successfully operate at medium-high temperatures.

**Figure 3:**
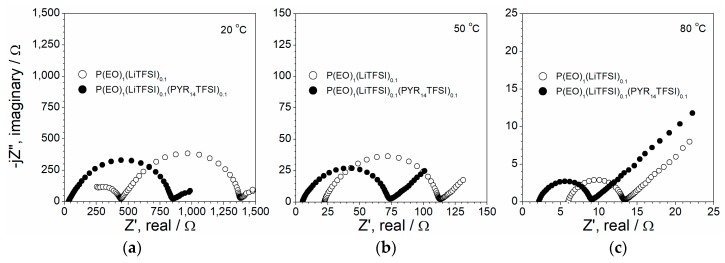
AC response of Li/P(EO)_1_(LiTFSI)_0.1_/Li and Li/P(EO)_1_(LiTFSI)_0.1_(PYR_14_TFSI)_0.1_/Li symmetrical cells at 20 °C (panel **a**), 50 °C (panel **b**) and 80 °C (panel **c**).

The compatibility with the lithium anode is a key parameter for applications as electrolyte separators in Li metal polymer batteries. Figure 3 compares the impedance plots of Li/P(EO)_1_(LiTFSI)_0.1_/Li and Li/P(EO)_1_(LiTFSI)_0.1_(PYR_14_TFSI)_0.1_/Li cells obtained at different temperatures. The AC responses are constituted by a semicircle, taking into account the overall Li/polymer electrolyte interfacial resistance (i.e., charge transfer + passive layer) [38], whereas the high frequency intercept with the real axis is associated with that of the electrolyte bulk [38]. It should be noted that, at 20 °C (panel a), the IL-free electrolyte shows a partial semicircle at high-medium frequencies, due to the relatively low conductivity of the sample P(EO)_1_(LiTFSI)_0.1_ [31]. Finally, the inclined straight-line, observed at low frequencies, is attributed to diffusive phenomena through the electrolyte (Warburg contribution) [38]. The impedance plots of Figure 3 clearly confirm how the incorporation of ionic liquid results in a significant decrease of the electrolyte resistance, especially from room to medium temperature, in agreement with the conductivity data reported in Table 1. However, a gain, even if moderate, in interface resistance is also detected. For instance, the P(EO)_1_(LiTFSI)_0.1_(PYR_14_TFSI)_0.1_ sample shows, at the interface with Li metal, a resistance of 10–11% lower (i.e., from 830 to 750 cm^2^ at 20 °C and from 7.0 to 6.3 cm^2^ at 80 °C) than that of the IL-free electrolyte (Table 1), in the whole investigated temperature range (20–80 °C). We can hypothesize that the ionic liquid improves the Li^+^ cation mobility at the electrolyte/lithium interface.

**Figure 4:**
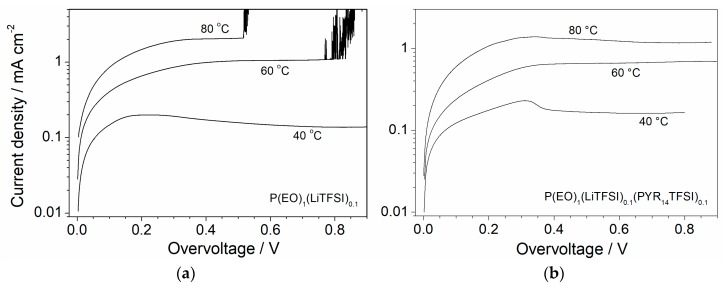
Current density vs. overvoltage curves obtained from potentiodynamic measurements carried out on Li/P(EO)_1_(LiTFSI)_0.1_/Li (panel **a**) and Li/P(EO)_1_(LiTFSI)_0.1_(PYR_14_TFSI)_0.1_/Li (panel **b**) cells at different temperatures.

Applications such as in automotives, smart grids, etc. require high power and for energy to be readily available; this means that this requires the battery system to be feasibly discharged and charged at high current rates without significantly depleting its performance. For instance, the increase of the current rate promotes the diffusive phenomena within the battery, thus lowering the content of the stored/delivered energy. In electrochemical cells, the redox process kinetics are generally much faster than the active species diffusion through the electrolyte separator. By increasing the current value, the matter transferring process becomes more and more predominant with respect to those at the interfaces with the electrodes. When the current flow through the cell achieves a limiting value, J_L_ (diffusion limiting current), the electrochemical processes are fully governed by the ion diffusion from the electrolyte bulk to the electrode surface and vice versa. Therefore, J_L_ is a key parameter for evaluating the feasibility of an electrolyte at high current rates. The limiting current value was determined as reported in Materials and Methods. For instance, linear sweep voltammetry tests were run (at 0.01 mV·s^−1^) on symmetrical Li/P(EO)_1_(LiTFSI)_0.1_/Li and Li/P(EO)_1_(LiTFSI)_0.1_(PYR_14_TFSI)_0.1_/Li cells at temperatures ranging from 40 to 80 °C. Figure 4 plots the current density values, recorded during the potentiodynamic measurements, as a function of the cell overvoltage. After an initial step increase, in which the electrolyte membrane shows a quasi-ohmic behavior, the current density is seen to progressively level off, likely associated with the establishment of a concentration gradient within the electrolyte membrane [46], around a time-stable value. Such a behavior indicates that the current density through the cell has reached the limiting value (J_L_), e.g., the ion conduction processes inside the electrolyte membrane are governed by diffusion phenomena (the concentration gradient extends through the overall electrolyte thickness). In Figure 4, it is shown how the J_L_ value remarkably increases with the operating cell temperature but is not affected by the presence of PYR_14_TFSI, i.e., from 0.13–017 to 1.2–2.0 mA·cm^−2^ (about one order of magnitude) in passing from 40 to 80 °C for both the IL-free (panel a) and the IL-containing (panel b) electrolyte. Therefore, the ionic liquid does not seem to reduce the diffusive phenomena through the PEO electrolyte. However, the current density of the P(EO)_1_(LiTFSI)_0.1_ sample, upon achieving the limiting value, quickly shows an abrupt feature during the potentiodynamic measurements at 60 °C and 80 °C (Figure 4a). This behavior, repeatedly confirmed by several (potentiodynamic) tests carried out on different Li/P(EO)_1_(LiTFSI)_0.1_/Li cells and never observed in the P(EO)_1_(LiTFSI)_0.1_(PYR_14_TFSI)_0.1_ sample, is ascribable to dendrite growth onto the Li electrode at current rates above 1 mA·cm^−2^. The results reported in Figure 4a suggest that the IL-free electrolyte is not able to sustain high current rates. Conversely, the ionic liquid plays a key role in improving the compatibility at the interface with the lithium anode, in particular when the cell is subjected to high current rates instead of in an open circuit condition as plotted in Figure 3. It is a plausible hypothesis that PYR_14_TFSI behaves as a protective agent towards the Li metal electrode, allowing the running of charge/discharge cycling tests at a high current density without appreciable degradation phenomena of the lithium anode. Once more, this confirms the beneficial effect resulting from ionic liquid incorporation on battery performance.

### 3.2. Composite Electrodes

**Figure 5:**
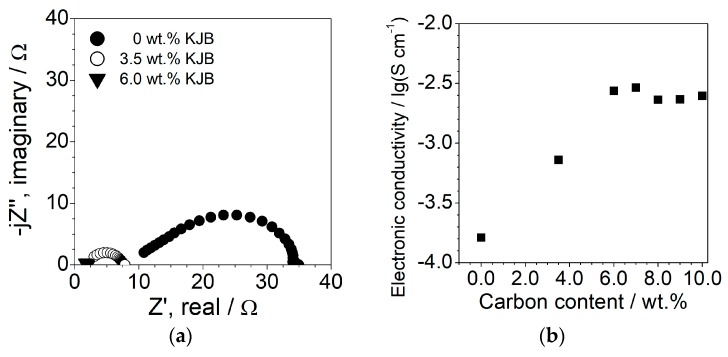
Panel (**a**): impedance plots of Al/LiFePO_4_ composite cathode/Al symmetrical cells at different carbon contents. Frequency range: 10 kHz–1 Hz. Temperature: 20 °C. Panel (**b**): electronic conductivity of LiFePO_4_ composite cathode as a function of the carbon content. Temperature: 20 °C.

The LiFePO_4_ electrode formulation was optimized in terms of carbon content in order to reach a good compromise between electronic conductor content and cathode performance. Therefore, electrode samples containing different carbon contents were prepared and investigated in terms of their electronic conductivity by impedance spectroscopy. The results are reported in Figure 5 as AC responses (panel a) and electronic conductivity vs. carbon content dependence (panel b). The impedance plot of the carbon-free sample (Figure 5a) is constituted by a semicircle (not starting from the axis origin) which does not display any capacitive contribution, indicating charge transfer at the interfaces with the Al° collectors [38]. This behavior—i.e., supporting electron conduction through the composite electrode—suggests the establishment of a three-dimensional network (percolation) formed by LiFePO_4_ particles and, therefore, electronic continuous pathways through the composite cathode [37]. It should be noted that the as-received active material is provided as superficially carbon-coated; this supports the not-very-low electronic resistance (given by the AC plot intercept with the real axis at low frequencies [38]) of the composite cathode (i.e., pure LiFePO_4_ material exhibits very low electronic conductivity [47]). The addition of KJB carbon around 3–4 wt. % results in a remarkable reduction of the semicircle diameter and a shifting of the low frequency intercept with the real axis towards smaller impedance values, highlighting a decrease of the electronic resistance of the cathode. At a KJB content equal to 6 wt. %, the semicircle practically reduces to a quasi-single point on the real axis, indicating that the electronic conductivity is largely overcome with respect to the ionic one (the electron and ion conductions through the polymer electrolyte are in parallel) of the polymer electrolyte incorporated within the electrode. In such a condition, the electronic resistance of the composite cathode is given by the distance of the “spot” response intercept with the real axis from the origin of the axes [38].

Figure 5b illustrates the electronic conductivity of the composite LiFePO_4_ cathode as a function of the carbon content. As evinced in Figure 5a, the electron conduction raises up to 7 wt. % of KJB with a gain of about 1.5 orders of magnitude. The further addition of carbon does not lead to any improvement of the electron transport properties, whereas it depletes the active material content and, therefore, the energy density of the composite cathode. Therefore, the KJB content in the LiFePO_4_ electrode was fixed to 7 wt. %.

### 3.3. Battery Tests at 80 °C

**Figure 6:**
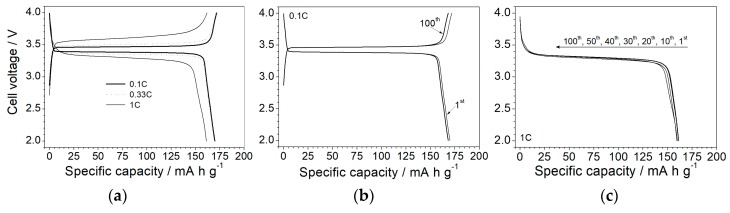
Panel (**a**): voltage vs. charge/discharge capacity profile of the 1st cycle of Li/P(EO)_1_(LiTFSI)_0.1_(PYR_14_TFSI)_0.1_/LiFePO_4_ polymeric cells at 80 °C and different current rates. Selected voltage vs. charge/discharge capacity profiles, obtained at 80 °C, of Li/P(EO)_1_(LiTFSI)_0.1_(PYR_14_TFSI)_0.1_/LiFePO_4_ cells at 0.1C (panel **b**) and 1C (panel **c**), respectively.

Upon investigation of the electrochemical performance, the P(EO)_1_(LiTFSI)_0.1_(PYR_14_TFSI)_0.1_ ionic liquid-based, polymer electrolyte was subjected to tests in Li/LiFePO_4_ cells at 80 °C. Figure 6a compares the voltage vs. capacity profile referring to the 1st charge–discharge cycle run at different current rates. A flat plateau, typical of the Li^+^ insertion/de-insertion process into the LiFePO_4_ active material [24,33,34], is observed (in the 3.3–3.6 V range) even at higher rates, with a coulombic efficiency close to 99%. This highlights that IL-incorporating Li/LiFePO_4_ cells are capable of maintaining the same voltage during almost the entire charge/discharge step. Only a 100 mV increase in ohmic drop is observed on passing from 0.1C through 1C. An initial capacity corresponding to the theoretical value (170 mA·h·g^−1^) is delivered up to the medium rate (0.33C) with just a moderate decrease at high current rates, i.e., more than 160 mA·h·g^−1^ (>94.1% of the theoretical capacity) are discharged at 1C. Figure 6b,c compares the voltage profiles of the selected charge/discharge cycles at 0.1C and 1C, respectively. It is worth noting that the excellent reproducibility of the battery performance, i.e., the profile feature and the delivered capacity, are practically unchanged after 100 consecutive cycles run (at 100% of deep of discharge, DOD) even at high current rates, which is not often reported for lab-scale, lithium metal polymer cells [24]. These results clearly show the very good reversibility of the Li^+^ intercalation process even under hard operating conditions in combination with an excellent compatibility at the electrolyte/electrode interface and negligible degradation phenomena occurring within the cell components. Such a performance score, however, can be achieved only through good manufacturing of the electrolyte/electrode components, i.e., high purity levels and careful optimization of the formulation, and of the full cells.

**Figure 7:**
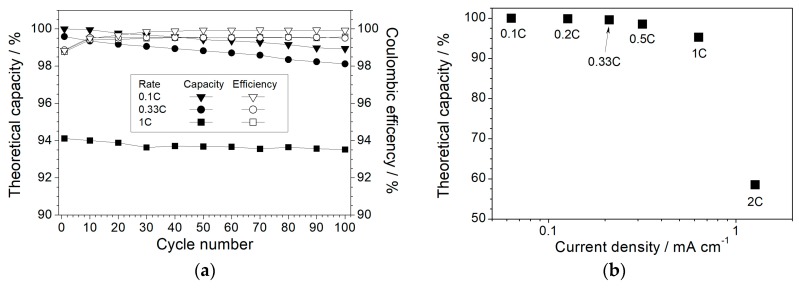
Capacity and coulombic efficiency vs. cycle number evolution at different current rates (panel **a**); and theoretical capacity vs. current density dependence (panel **b**) of Li/P(EO)_1_(LiTFSI)_0.1_(PYR_14_TFSI)_0.1_/LiFePO_4_ polymeric cells at 80 °C. The corresponding current rates are also reported.

The cycling performance of the Li/P(EO)_1_(LiTFSI)_0.1_(PYR_14_TFSI)_0.1_/LiFePO_4_ solid-state cells, tested at 80 °C and different current rates, is depicted in Figure 7a. An excellent capacity retention (as also evinced in Figure 6b,c) with a coulombic efficiency quickly leveling above 99.5% (100% at 0.1C) is recorded even at higher rates, i.e., more than 99.5% and 94% of theoretical capacity are initially delivered at 0.33C and 1C, respectively, with a very modest decay (>98% and 93.6%, respectively) after 100 consecutive cycles. This corresponds to a capacity fading around 0.005% per cycle and, in conjunction with the very good charge/discharge efficiency, once more highlights a highly reversible lithiation process in combination with the high purity level and high compatibility of the P(EO)_1_(LiTFSI)_0.1_(PYR_14_TFSI)_0.1_ polymer electrolyte towards electrodes, in particular with the lithium metal anode, even at high current rates. Also, it should be noted that very clean lithium metal tapes were used for the cell manufacturing in order to obtain an optimal Li/electrolyte interface. Especially, we would like to point out the absence of dendrite growth on the Li electrode during prolonged cycling tests run also at 1C, i.e., very rarely encountered in lithium metal polymer batteries operating at medium-high temperatures under high rates [24]. These experimental data, in rather good agreement with the results derived from potentiodynamic measurements depicted in Figure 4, once more demonstrate that the incorporation of ionic liquids such as PYR_14_TFSI significantly improves the PEO electrolyte interface with the lithium anode, allowing high current rates to be sustained for prolonged cycling tests without appreciably depleting the cell performance.

The capacity vs. current density dependence (80 °C) is plotted in Figure 7b, which evinces a very good rate capability. Above 94% of the theoretical value is still obtained at 1C, supporting an excellent rate capability up to 1C, i.e., corresponding to about 0.7 mA·cm^−2^, which represents a very interesting current value for an all-solid-state polymer electrolyte. A further increase of the current rate up to 2C, i.e., around 1.4 mA·cm^−2^, leads to a reduction of the delivered capacity which levels off at 57% of the theoretical value. This behavior, ascribable to diffusive phenomena within the electrolyte separator, is in good agreement with the results obtained by the potentiodynamic measurements (Figure 3b), which indicates that above a current density of about 1.2 mA·cm^−2^ (determined as J_L_ value), the electrochemical processes through the cell are controlled by the diffusive phenomena occurring within the polymer electrolyte. However, despite the capacity decay due to the operating current density exceeding the limiting value, the Li/P(EO)_1_(LiTFSI)_0.1_(PYR_14_TFSI)_0.1_/LiFePO_4_ cells are still able to deliver about 100 mA·h·g^−1^ at a rate as high as 2C (about 1.4 mA·cm^−2^), i.e., representing a remarkable capacity value for an all-solid-state polymer electrolyte.

The battery performance of the P(EO)_1_(LiTFSI)_0.1_(PYR_14_TFSI)_0.1_ electrolyte, detected at 80 °C in Li/LiFePO_4_ cells, is compared with that of other lithium-conducting, ionic liquid-free, PEO membranes, recorded in Li/LiFePO_4_ and Li/V_2_O_5_ systems at temperatures from 90 °C to 100 °C [18,22,23]. The data, reported in Table 2, show how appreciable capacities, i.e., from 70 to 96% of the cell theoretical value, are delivered only at low-medium rates (0.2C–0.33C). However, a capacity decay down to 45–60% of the theoretical value is observed after 100 consecutive charge/discharge cycles, with a fading corresponding to 0.26–0.36% per cycle. Conversely, very modest capacities, i.e., from 8 to 14% of the theoretical value, are obtained when the current rate is increased up to 0.8C–1C. From the data illustrated in Figure 6 and Figure 7 and Table 2, it is evident how, at medium-high temperatures, the PYR_14_TFSI-incorporating lithium polymer batteries behave much better in terms of their delivered capacity and cycling performance than the IL-free ones. For instance, the addition of suitable ionic liquid is able to largely improve the performance of the LPBs not only at ambient or near ambient conditions, as previously reported in the literature [18,20,22,23], but even at medium-high temperatures. Therefore, the PEO-LiTFSI-PYR_14_TFSI Li^+^-conducting membranes are very promising candidates as electrolyte separator systems for all-solid-state lithium polymer batteries operating around 100 °C.

## 4. Conclusions

PEO-LiTFSI Li^+^-conducting membranes, containing the PYR_14_TFSI ionic liquid, were prepared and studied to be addressed as electrolyte separators for all-solid-state lithium polymer batteries operating at medium-high temperatures. A solvent-free procedure was designed to prepare the PEO-LiTFSI-PYR_14_TFSI electrolytes. These ternary systems have shown remarkably improved thermal, ion transport and interfacial properties with respect to the ionic liquid-free electrolytes. Wide electrochemical stability was observed even at medium-high operating temperatures. In particular, the ionic liquid-based PEO electrolytes are able to sustain high current rates without any appreciable lithium anode degradation, which is not allowed in binary ionic liquid-free, PEO-LiTFSI systems, thus enabling their use in battery systems operating at 80 °C or above and high current rates. Battery tests carried out at 80 °C in Li/LiFePO_4_ polymeric systems have shown excellent cycling behavior and capability retention at high current rates, e.g., more than 93.6% of the theoretical capacity (i.e., 99.5% of the initial value) is still delivered after 100 cycles run at 1C with a coulombic efficiency close 100%. This performance largely exceeds that of analogous, ionic liquid-free, polymer lithium batteries at the same operating conditions, nominating the PEO-LiTFSI-PYR_14_TFSI ternary system as an electrolyte separator for medium-high temperature lithium polymer batteries. It is worth highlighting the absence of dendrite growth on the Li anode during prolonged cycling tests even at high current rates, which is very often not observed in lithium metal polymer batteries.

## Figures and Tables

**Table 1:** Ionic conductivity and Li anode/polymer electrolyte interface resistance of the poly(ethyleneoxide) (P(EO))_1_(LiTFSI)_0.1_ and P(EO)_1_(LiTFSI)_0.1_(PYR_14_TFSI)_1_ polymer electrolytes at different temperatures. (*) from ref. [31].

Polymer Electrolyte Sample	Ionic Conductivity/S·cm^−1^			
	−20 °C	20 °C	50 °C	80 °C
P(EO)_1_(LiTFSI)_0.1_ (*)	1.1 × 10^−9^	1.3 × 10^−6^	2.2 × 10^−4^	8.4 × 10^−4^
P(EO)_1_(LiTFSI)_0.1_(PYR_14_TFSI)_0.1_ (*)	9.7 × 10^−7^	1.1 × 10^−4^	7.9 × 10^−4^	1.9 × 10^−3^
	**Li/PE Interfacial Resistance/cm^2^**			
P(EO)_1_(LiTFSI)_0.1_	n.a.	830 ± 80	82 ± 8	7.0 ± 0.7
P(EO)_1_(LiTFSI)_0.1_(PYR_14_TFSI)_0.1_	n.a.	750 ± 70	65 ± 6	6.3 ± 0.6

**Table 2:** Summary of the battery performance of the P(EO)_1_(LiTFSI)_0.1_(PYR_14_TFSI)_1_ polymer electrolyte at 80 °C compared with that of lithium-conducting, ionic liquid-free, PEO membranes at medium-high temperatures. (a) From reference [22]; (b) from reference [23]; (c) from reference [18]; (d) this work.

Polymer Electrolyte Sample	Battery System	T/°C	Current Density/mA·cm^−2^	Percent of Theoretical Capacity/%
P(EO)_1_(LiCF_3_SO_3_)_0.05_ (a)	Li/Cu_0.1_V_2_O_5_	90	0.1 (0.2C)	96 (1st) → 60 (100th)
P(EO)_1_(LiBETI)_0.05_ (b)	Li/V_2_O_5_	90	0.24 (0.33C)	70 (1st) → 45 (100th)
P(EO)_1_(LiBETI)_0.05_ (b)	Li/V_2_O_5_	90	0.72 (1C)	14 (1st)
P(EO)_1_(LiCF_3_SO_3_)_0.03_ + 5 wt. % SiO_2_ (c)	Li/LiFePO_4_	100	0.2 (0.2C)	82 (1st) → 47 (100th)
P(EO)_1_(LiCF_3_SO_3_)_0.03_ + 5 wt. % SiO_2_ (c)	Li/LiFePO_4_	100	0.8 (0.8C)	8 (1st)
P(EO)_1_(LiTFSI)_0.1_(PYR_14_TFSI)_0.1_ (d)	Li/LiFePO_4_	80	0.7 (1C)	94.1 (1st) → 93.6 (100th)

## References

[1] Notter D.A., Gauch M., Widmer R., Wäger P., Stamp A., Zah R., Althaus H.-J. (2010). Contribution of Li-ion batteries to the environmental impact of electric vehicles. Environ. Sci. Technol..

[2] Yang H., Amiruddin S., Bang H.J., Sun Y.K., Prakash J. (2006). A review of Li-ion cell chemistries and their potential use as hybrid electric vehicles. J. Ind. Eng. Chem..

[3] Nair J.R., Chiappone A., Destro M., Jabbour L., Meligrana G., Gerbaldi C. (2012). UV-induced radical photo-polymerization: A smart tool for preparing polymer electrolyte membranes for energy storage devices. Membranes.

[4] Hu P., Duan Y., Hu D., Qin B., Zhang J., Wang D., Liu Z., Cui G., Chen L. (2015). Rigid−flexible coupling high ionic conductivity polymer electrolyte for an enhanced performance of LiMn_2_O_4_/graphite battery at elevated temperature. ACS Appl. Mater. Int..

[5] Colò F., Bella F., Nair J.R., Gerbaldi C. (2017). Light-cured polymer electrolytes for safe, low-cost and sustainable sodium-ion batteries. J. Power Sources.

[6] Dyartanti E.R., Purwanto A., Widiasa I.N., Susanto H. (2018). Ionic conductivity and cycling stability improvement of PVdF/nano-clay using PVP as polymer electrolyte membranes for LiFePO_4_ batteries. Membranes.

[7] Lia H., Lia M., Siyala S.H., Zhua M., Lana J.-L., Suia G., Yua Y., Zhonga W., Yang X. (2018). A sandwich structure polymer/polymer-ceramics/polymer gel electrolytes for the safe, stable cycling of lithium metal batteries. J. Membr. Sci..

[8] Zhao Y., Zhang Y., Gosselink D., Long Doan T.N., Sadhu M., Cheang H.J., Chen P. (2012). Polymer electrolytes for lithium/sulfur batteries. Membranes.

[9] Yang M., Hou J. (2012). Membranes in lithium ion batteries. Membranes.

[10] Spotnitz R., Franklin J. (2003). Abuse behavior of high-power, lithium-ion cells. J. Power Sources.

[11] Abraham D.P., Roth E.P., Kostecky R., McCarthy K., MacLaren S., Doughty D.H. (2006). Diagnostic examination of thermally abused high-power lithium-ion cells. J. Power Sources.

[12] Bandhauer T.M., Garimella S., Fuller T.F. (2011). A critical review of thermal issues in lithium-ion batteries. J. Electrochem. Soc..

[13] Armand M., Chabagno J.M., Duclot M., Vashitshta P., Mundy J.N., Shenoy G.K. (1979). Poly-ethers as solid electrolytes. Fast Ion Transport in Solids. Electrodes and Electrolytes.

[14] Gray F.M. (1997). Polymer Electrolytes.

[15] Lightfoot P., Metha M.A., Bruce P.G. (1993). Crystal structure of the polymer electrolyte Poly(ethylene oxide)_3_: LiCF_3_SO_3_. Science.

[16] Vincent C.A., Scrosati B. (1993). Modern Batteries. An Introduction to Electrochemical Power Sources.

[17] Gray F.M., Armand M., Osaka T., Osaka T., Datta M. (2000). Energy Storage System for Electronics.

[18] Appetecchi G.B., Croce F., Hassoun J., Scrosati B., Salomon M., Cassel F. (2003). Hot-pressed, solvent-free, nanocomposite, PEO-based electrolyte membranes. II. All-solid, Li/LiFePO_4_ polymer batteries. J. Power Sources.

[19] Appetecchi G.B., Scaccia S., Passerini S. (2000). Investigation on the stability of the lithium-polymer electrolyte interface. J. Electrochem. Soc..

[20] Appetecchi G.B., Alessandrini F., Duan R.G., Arzu A., Passerini S. (2001). Electrochemical testing of industrially produced PEO-based polymer electrolytes. J. Power Sources.

[21] Appetecchi G.B., Henderson W., Villano P., Berrettoni M., Passerini S. (2001). PEO-LiN(SO_2_CF_2_CF_3_)_2_ polymer electrolytes. I. XRD, DSC and ionic conductivity characterization. J. Electrochem. Soc..

[22] Appetecchi G.B., Alessandrini F., Carewska M., Caruso T., Prosini P.P., Scaccia S., Passerini S. (2001). Investigation on the lithium polymer electrolyte batteries. J. Power Sources.

[23] Villano P., Carewska M., Appetecchi G.B., Passerini S. (2002). PEO-LiN(SO_2_CF_2_CF_3_)_2_ polymer electrolytes. III. Tests in batteries. J. Electrochem. Soc..

[24] Passerini S., Montanino M., Appetecchi G.B., Mittal V. (2013). Lithium polymer batteries based on ionic liquids. Polymers for Energy Storage and Conversion.

[25] Chiappe C., Pieraccini D. (2005). Ionic liquids: Solvent properties and organic reactivity. J. Phys. Org. Chem..

[26] Rogers J.R.D., Seddon K.R. (2002). Ionic Liquids: Industrial Application to Green Chemistry.

[27] Ohno H. (2005). Electrochemical Aspects of Ionic Liquids.

[28] Shin J.-H., Henderson W.A., Passerini S. (2003). Ionic liquids to the rescue? Overcoming the ionic conductivity limitations of polymer electrolytes. Electrochem. Commun..

[29] Shin J.-H., Henderson W.A., Appetecchi G.B., Alessandrini F., Passerini S. (2005). Recent developments in the ENEA lithium metal battery project. Electrochim. Acta.

[30] Shin J.-H., Henderson W.A., Tizzani C., Passerini S., Jeong S.-S., Kim K.-W. (2006). Characterization of solvent-free polymer electrolytes consisting of ternary PEO-LiTFSI-PYR_14_TFSI. J. Electrochem. Soc..

[31] Kim G.-T., Appetecchi G.B., Alessandrini F., Passerini S. (2007). Solvent-free, PYR1ATFSI ionic liquids-based ternary polymer electrolyte systems. I. Electrochemical characterization. J. Power Sources.

[32] Kim G.-T., Appetecchi G.B., Carewska M., Joost M., Balducci A., Winter M., Passerini S. (2010). UV cross-linked, lithium-conducting ternary polymer electrolytes containing ionic-liquids. J. Power Sources.

[33] Appetecchi G.B., Kim G.-T., Montanino M., Alessandrini F., Passerini S. (2011). Room temperature lithium polymer batteries based on ionic liquids. J. Power Sources.

[34] Kim G.-T., Jeong S.-S., Xue M.-Z., Balducci A., Winter M., Passerini S., Alessandrini F., Appetecchi G.B. (2012). Development of ionic liquid-based lithium battery prototypes. J. Power Sources.

[35] Montanino M., Alessandrini F., Passerini S., Appetecchi G.B. (2013). Water-based synthesis of hydrophobic ionic liquids for high-energy electrochemical devices. Electrochim. Acta.

[36] De Francesco M., Simonetti E., Giorgi G., Appetecchi G.B. (2017). About purification route of hydrophobic ionic liquids. Challenges.

[37] Appetecchi G.B., Carewska M., Alessandrini F., Prosini P.P., Passerini S. (2000). Characterization of PEO-based composite cathodes. I. Morphological, thermal, mechanical and electrical properties. J. Electrochem. Soc..

[38] MacDonald J.R. (1987). Impedance Spectroscopy.

[39] Boukamp B.A. (1986). A package for impedance/admittance data analysis. Solid State Ion..

[40] Boukamp B.A. (1986). A nonlinear least squares fit procedure for analysis of immittance data of electrochemical systems. Solid State Ion..

[41] Simonetti E., Carewska M., Di Carli M., Moreno M., De Francesco M., Appetecchi G.B. (2017). Towards improvement of the electrochemical properties of ionic liquid-containing polyethylene oxide-based electrolytes. Electrochim. Acta.

[42] Appetecchi G.B., Montanino M., Carewska M., Moreno M., Alessandrini F., Passerini S. (2011). Chemical-physical properties of bis(perfluoroalkylsulfonyl)imide anion-based ionic liquids. Electrochim. Acta.

[43] Henderson W.A., Passerini S. (2004). Phase behavior of ionic liquid—LiX mixtures:  pyrrolidinium cations and TFSI^-^ anions. Chem. Mater..

[44] Passerini S., Scrosati B. (1994). Characterization of nonstoichiometric nickel oxide thin-film electrodes. J. Electrochem. Soc..

[45] Randstrom S., Montanino M., Appetecchi G.B., Lagergren C., Moreno A., Passerini S. (2008). Effect of water and oxygen traces on the cathodic stability of *N*-alkyl-*N*-methylpyrrolidinium bis(trifluoromethanesulfonyl)imide. Electrochim. Acta.

[46] Bard A.J., Faulkner L.R. (1980). Electrochemical Methods.

[47] Wang C., Hong J. (2007). Ionic/electronic conducting characteristics of LiFePO_4_ cathode materials. The determining factors for high rate performance. J. Electrochem. Soc..

